# Bioinformatics analysis revealing prognostic significance of TIMP2 gene in breast cancer

**DOI:** 10.1097/MD.0000000000027489

**Published:** 2021-10-22

**Authors:** Wen-Quan Chen, Su-Jin Yang, Wen-Xiu Xu, Fei Deng, Dan-Dan Wang, Jin-Hai Tang

**Affiliations:** a Department of General Surgery, the First Affiliated Hospital with Nanjing Medical University, 300 Guanzhou Road, Nanjing, China; b Department of General Surgery, Pukou Branch of Jiangsu Province Hospital, 166 Shanghe Road, Nanjing, China.

**Keywords:** bioinformatics analysis, biomarker, breast cancer, TIMP2

## Abstract

Tissue inhibitor of metalloproteinases 2 (TIMP2) is a member of the TIMP gene family. Accumulated evidence indicates that TIMP2 plays a significant role in various tumor processes including cell growth, apoptosis, invasion, and metastasis. However, the expression patterns and exact roles of TIMP2 had not been elucidated in breast cancer. In our research, we evaluated the expression and prognostic value of TIMP2 in breast cancer through analyzing various databases including Oncomine, bc-GenExMiner, PrognoScan, UCSC Xena, Kaplan–Meier Plotter, and PPI network. The results showed that TIMP2 was down-regulated in various breast cancer subtypes. Additionally, TIMP2 was significantly associated with age, estrogen receptor status, basal-like group, triple-negative breast cancer, PAM50 subtypes, and RSSPC subtypes. Also, the expression of TIMP2 was related to overall survival with different clinical characteristics. We analyzed the co-expressed genes with TIMP2 and interaction information with other proteins. These results disclosed that TIMP2 might serve as a potential target and prognostic biomarker in breast cancer. However, additional research is required to demonstrate our findings and motivate the clinical importance of TIMP2 in breast cancer.

## 1 Introduction

Breast cancer remains the most common cancer and the leading cause of cancer-related death among women worldwide. It had been estimated that there were 268,600 new cases and 41,760 deaths of breast cancer worldwide in 2019 and the incidence and mortality are rapidly increasing year by year.^[1]^ Breast cancer is a heterogeneous tumor and is different in clinical symptoms, type of pathology, hormone receptor, Herceptin receptor, prognosis, epigenetics, and response to therapy. Growing evidence showed that multiple genes and signaling pathways were involved in breast cancer. Therefore, it is valuable to find a new molecular marker severed as a therapeutic target, diagnostic marker, progression, and prognosis for breast cancer.

Tissue inhibitor of metalloproteinases 2 (TIMP2), a secreted 21 kDa multifunctional protein and a member of TIMP gene family, could inhibit matrix metalloproteinases (MMPs), which was associated with degradation of extracellular matrix and was a key role in cancer metastasis. TIMPs, including TIMP2, were demonstrated that had antitumor activity by mediating the activation of cell signaling pathways. Increasing researches disclosed that TIMP2 was associated with advanced stage, metastasis, and poor survival of cancers, such as breast cancer, cervical cancer, colorectal carcinoma, osteosarcoma, and so on.^[2–5]^ However, the roles of TIMP2 in breast cancer remain unexplored.

In this research, we used several large online databases to perform a bioinformatics analysis which was associated with TIMP2 in the clinical characteristics and survival data of breast cancer. This research aims to evaluate the prognostic significance of TIMP2 in breast cancer treatment.

## 2 Materials and methods

### 2.1 Oncomine

The Oncomine(www.oncomine.org) is a gene expression array dataset and an accessible, public, online cancer microarray database, which could facilitate research from genome-wide expression analyses.^[6]^ The Oncomine is performed to evaluate the mRNA level of TIMP2 during different cancers vs normal tissues and in various types of breast cancers. The thresholds were restricted as *P* vaule ≤1E-4, gene rank top 10%, and fold-change ≥2-fold. In addition, gene co-expression with TIMP2 was analyzed in this database.

### 2.2 Breast cancer gene-expression miner

The breast cancer gene-expression miner (bc-GenExMiner) (http://bcgenex.centregauducheau.fr/, v4.2), a statistical mining tool, offered the potentiality to evaluate prognostic information of various genes in breast cancer.^[7,8]^ We used the database to visually compare TIMP2 expression in different groups during age, estrogen receptor (ER), progestogen receptor (PR), human epidermal growth factor receptor 2 (Her-2), nodal status, SBR, NPI, triple-negative status, basal-like status, PAM50 subtypes, and RSSPC subtypes. Moreover, we assessed the correlation of TIMP2 and fibronectin 1 (FN1). Last update was January 2019.

### 2.3 PrognoScan

The PrognoScan (http://www.prognoscan.org/) is a new database for meta-analysis of prognostic of genes and shows a relationship between patient prognosis and gene expression like disease-free survival and overall survival (OS).^[9]^

### 2.4 Kaplan–Meier plotter

The Kaplan–Meier plotter (www.kmplot.com) is an online database containing microarray gene expression data and survival information. In this research, Kaplan–Meier plotter evaluated the prognostic value of expression of TIMP2. The OS of breast cancer patients were determined by age, size of tumor, node status, metastasis, ER, PR, Her-2, and stage.

### 2.5 University of California Santa Cruz Xena database

The University of California Santa Cruz Xena (UCSC Xena) (http://xena.ucsc.edu/) provided interactive online visualization of seminal cancer genomics datasets from several datasets including The Cancer Genome Atlas. Xena supports virtually functional genomics data including gene expression.

### 2.6 Protein interaction network and pathway interaction analysis building

Protein interaction network networks information was evaluated through String version 11.0 (https://string-db.org/), which was a web tool and to discover protein interactions information of TIMP2.^[10]^

All data were derived from public databases, so ethical approval was not necessary.

## 3 Results

### 3.1 The expression of TIMP2 gene in breast cancer patients

**Figure 1:**
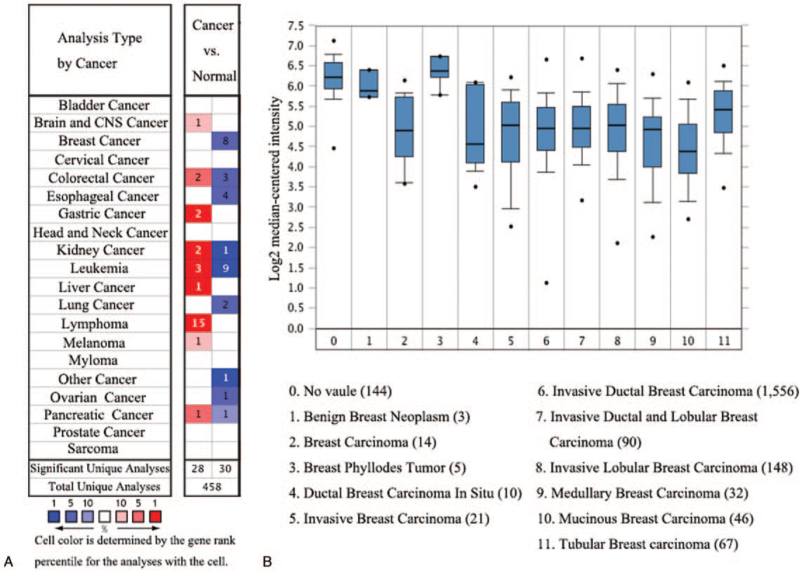
A) Expression of TIMP2 in 20 common cancers vs paired normal tissues through Oncomine database. B) Expression of TIMP2 in various types of breast cancers. TIMP2 = tissue inhibitor of metalloproteinases 2.

**Figure 2:**
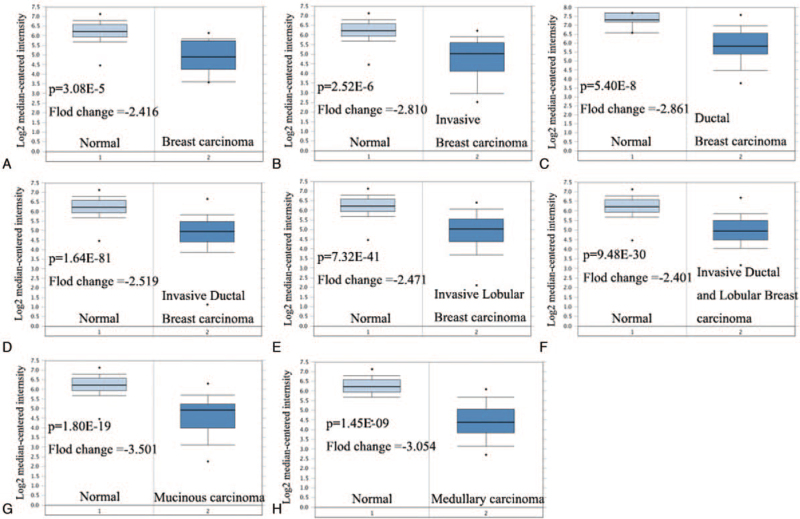
Box plot comparing TIMP2 expression in normal tissues vs breast cancer patients from Oncomine database. A) Breast carcinoma; B) invasive breast carcinoma; C) ductal breast cancer; D) invasive ductal breast carcinoma; E) invasive lobular breast carcinoma; F) invasive ducal and lobular breast carcinoma; G) mucinous carcinoma; and H) medullary carcinoma. TIMP2 = tissue inhibitor of metalloproteinases 2.

At first, we compared the mRNA expression of TIMP2 in 20 types of cancer with that in normal tissues through Oncomine databases. The analysis showed that the expression of TIMP2 was down-regulated in breast cancer and ovarian cancer, and up-regulated in gastric cancer, lymphoma, and melanoma (Fig. 1A). Especially, the expression of TIMP2 was significantly dysregulated in different types of breast cancer, like benign breast neoplasm, breast carcinoma, ductal breast carcinoma in situ, breast phyllodes tumor, invasive breast carcinoma, invasive ductal breast carcinoma, invasive lobular breast carcinoma, medullary breast carcinoma, mucinous breast carcinoma, and tubular breast carcinoma (Fig. 1B). In detail, Oncomine analysis also showed that TIMP2 was significantly down-regulated in breast carcinoma, ductal breast carcinoma, invasive breast carcinoma, invasive lobular breast carcinoma, invasive ductal and lobular breast carcinoma, mucinous breast carcinoma, and medullary breast carcinoma (Fig. 2A–H, Table 1)

**Table 1:** The expression of TIMP2 in different subtypes of breast cancer and compared normal tissues through the Oncomine database.

Breast cancer subtype	*P* vaule	*t* test	Fold change	Sample
Breast carcinoma	3.08E-05	−0.566	−2.416	14
Invasive breast carcinoma	2.52E-06	−6.065	−2.810	21
Ductal breast carcinoma	5.40E-8	−7.521	−2.861	40
Invasive ductal breast carcinoma	1.64E-81	−30.227	−2.471	1556
Invasive lobular breast carcinoma	7.32E-41	−16.372	−2.471	148
Invasive ductal and lobular breast carcinoma	9.48E-30	−14.551	−2.401	90
Mucinous breast carcinoma	1.80E-19	−13.670	−3.501	46
Medullary breast carcinoma	1.45E-10	−8.780	−3.054	32

### 3.2 TIMP2 expression and clinical parameters in breast cancer patients

**Figure 3:**
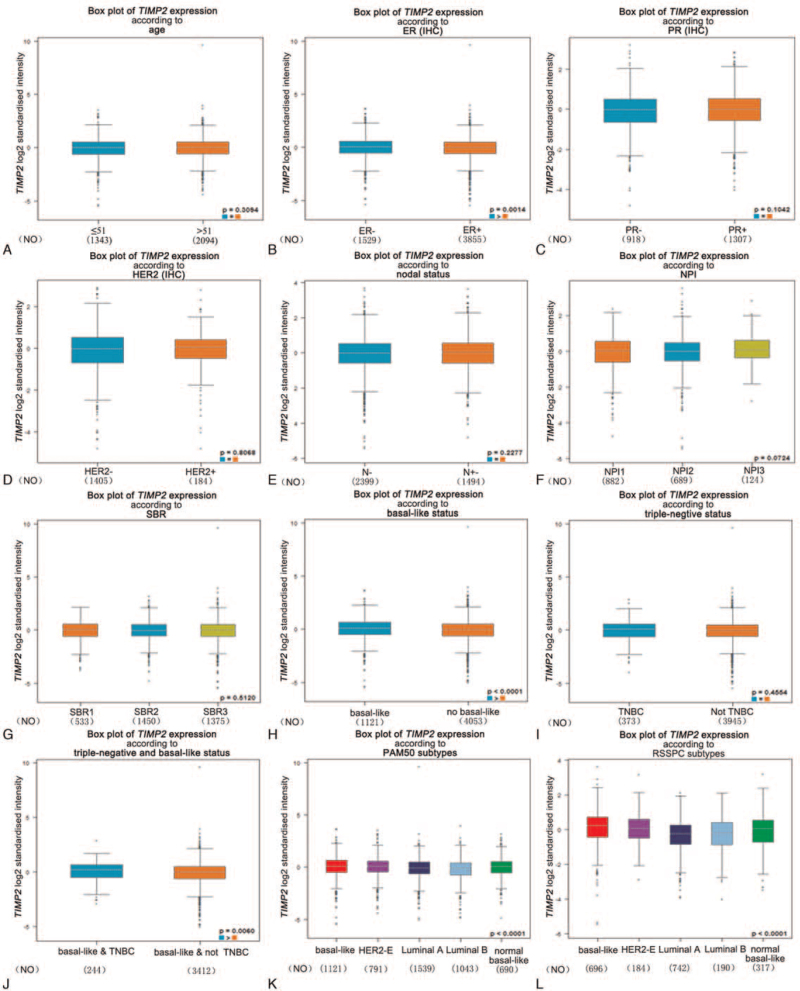
Box plot evaluating TIMP2 expression among different groups of patients based on clinical parameters using bc-GenExMiner software. A) Age; B) ER; C) PR; D) Her-2; E) Nodal status; F) NPI; G) SBR; H) basal-like status; I) triple-negative breast cancer; J) triple-negative and basal-like status; K) PAM50 subtypes; and L) RSSPC subtypes. bc-GenExMiner = Breast cancer Gene-Expression Miner, ER = estrogen receptor, Her-2 = human epidermal growth factor receptor 2, PR = progestogen receptor, TIMP2 = tissue inhibitor of metalloproteinases 2.

For further, we evaluated the expression of TIMP2 among different clinical parameters in breast carcinoma through bc-GenExMiner software. For age, it was not statistically significant in groups of ≤51 and >51 years (Fig. 3A, Table 2). TIMP2 was significantly up-regulated in ER-negative group (*P* = .0014), basal-like group and basal-like (*P* < .0001), and triple negative breast cancer group (*P* = .0060) (Fig. 3B, H, and J, Table 2). In addition, TIPM2 had no significant difference in PR status, Her-2 status, lymph node status, NPI, SBR, and triple-negative status (Fig. 3C, D, E, F, I, and 3G, Table 2). Regarding PAM50 subtypes (*P* < .0001) and RSSPC subtypes (*P* < .0001), there was statistical significance in the expression of TIMP2 (Fig. 3K and L, and Table 2).

**Table 2:** Relationships between the expression of TIMP2 and clinical characteristics of breast cancer patients using the bc-GenExMiner database.

Variables	Number of patients	*P* vaule
Age		
≤51	1343	.3094
>51	2094	
ER		**.0014**
Negative	1529	
Positive	3855	
PR		.1042
Negative	918	
Positive	1307	
Her-2		.8068
Negative	1405	
Positive	184	
Lymph node		.2277
Negative	2399	
Positive	1494	
NPI		.0724
1	882	
2	689	
3	124	
SBR		.512
1	533	
2	1450	
3	1375	
Basal-like status		
Yes	1121	**<.0001**
No	4063	
Basal-like and TNBC		
Yes	244	.0060
No	3412	
RSSPC subtypes		**<.0001**
Basal-like	696	
Her-2	184	
Luminal A	742	
Luminal B	190	
Normal breast-like	317	
PAM50 subtypes		**<.0001**
Basal-like	1121	
Her-2	791	
Luminal A	1539	
Luminal B	1043	
Normal breast-like	690	

### 3.3 TIMP2 expression and prognosis in breast cancer patients

**Figure 4:**
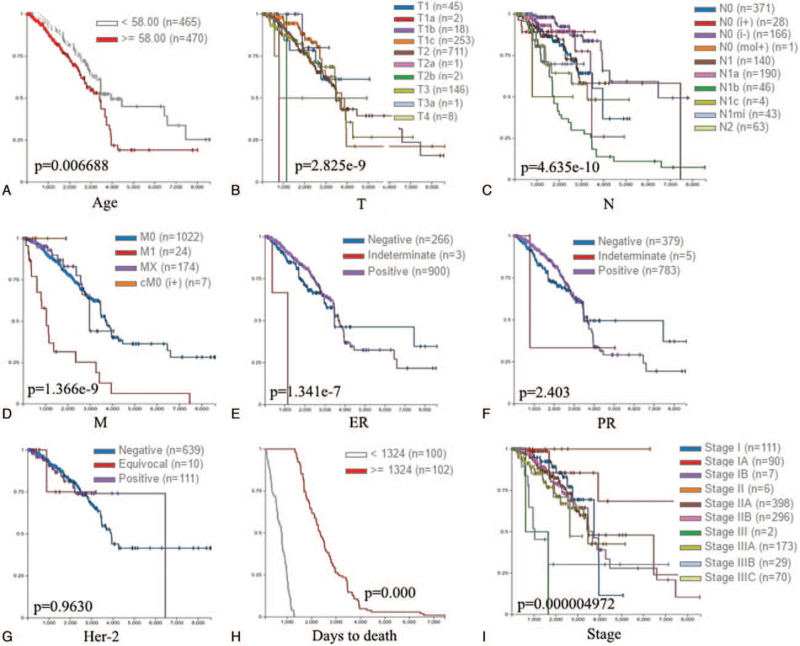
Survival curve evaluating the prognosis value of TIMP2 in different characteristics of breast cancer using UCSC Xena. A) Age; B) size of tumor (T); C) lymph node (N); D) metastasis (M); E) ER; F) PR; G) Her-2; H) days to death; and I) stage. ER = estrogen receptor, Her-2 = human epidermal growth factor receptor 2, M = mestasis, N = node, PR = progestogen receptor, T = the size of tumor, TIMP2 = tissue inhibitor of metalloproteinases 2, UCSC Xena = University of California Santa Cruz Xena database.

**Figure 5:**
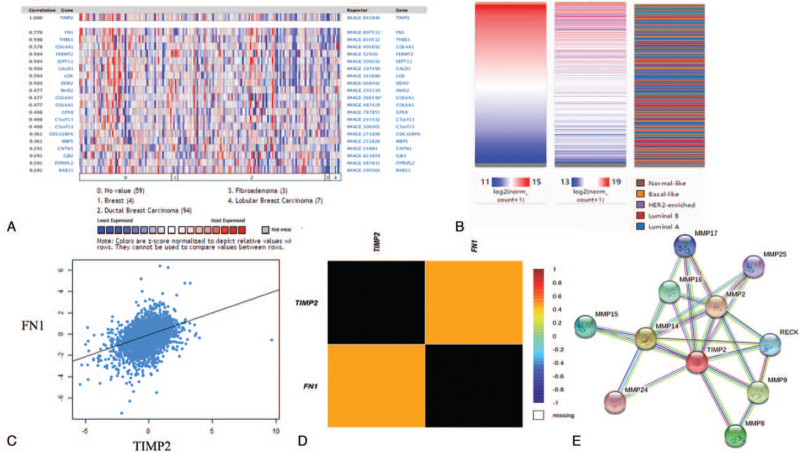
Co-expression of TIMP2 gene. A) TIMP2 co-expression of genes identified by the Oncomine database; B) heat map of TIMP2 and FN1 expression in various subtypes in TCGA database by UCSC Xena web-based tool; C) and D) co-expression TIMP2 and FN1 expression in breast cancer by bc-GenExMiner software; and E) Spring software showed the co-expression of TIMP2. bc-GenExMiner = Breast cancer Gene-Expression Miner, FN1 = Fibronectin 1, TIMP2 = tissue inhibitor of metalloproteinases 2, UCSC Xena = University of California Santa Cruz Xena database.

UCSC Xena has survival analyses that are complete with *P* values, custom time variable cutoff and multiple survival endpoints. Further, we evaluated the prognostic value of TIMP2 by UCSC Xena. The Kaplan–Meier survival curves were shown in Figure 4 and Table 3. High expression of TIMP2 was correlated to poor overall survival (OS) in ≥58 years group compared to <58 years group (*P* = .0006688) (Fig. 4A and Table 3). In different TNM stages, the expression of TIMP2 had a statistical significance in size of tumor (T), nodal state (N), and metastasis (M) (*P* = 2.825e-9, *P* = 4.635e-10, and *P* = 1.366e-9) (Fig. 4B, C, and D, and Table 3). About ER status, high-expressed TIMP2 was related to poor OS in ER-positive group compared to ER-negative group (*P* = 1.341e-7) (Fig. 4E and Table 3). However, there were no significant differences in PR and Her-2 status (Fig. 4F and G, and Table 3). Furthermore, the expression of TIMP2 had a significant difference with days to death and stages (*P* = .000 and *P* = .000004972) (Fig. 4H and I, and Table 3). Co-expression of TIMP2 gene. Finally, we further investigated the co-expression of TIMP2 through the Oncomine database. The co-expression profile of TIMP2 was confirmed with a larger cluster of genes across different breast diseases (Fig. 5A). After analyzing the data in the TCGA database by the UCSC Xena web-based tool, we also showed a positive correlation between the expression of TIMP2 and FN1, as shown in the heatmap (Fig. 5B). Data mining using the bc-GenExMiner software revealed a positive correlation between TIMP2 and FN1 expression (Fig. 5C and D). The String database showed the interactions information of TIMP2 with other proteins using all publicly available sources (Fig. 5E)

**Table 3:** The prognostic value of TIMP2 in various clinical characteristics.

Variables	Number of patients	Log-rank test statistcis	*P* vaule
Age		11.57	**.0006688**
≤58	465		
>58	470		
T		58.32	**2.825e-9**
T1	45		
T1a	2		
T1b	18		
T1c	253		
T2	711		
T2a	1		
T2b	2		
T3	146		
T3a	1		
T4	8		
N		62.39	**4.635e-10**
N0	371		
N0 (i+)	28		
N0 (i−)	166		
N0 (mol+)	1		
N1	140		
N1a	190		
N1b	46		
N1c	4		
N1mi	43		
N2	63		
M		44.20	**1.366e-9**
M0	1022		
M1	24		
Mx	174		
cM0 (i+)	7		
ER		31.65	**1.341e-7**
Negative	266		
Indeterminate	3		
Positive	900		
PR		2.403	.3008
Negative	379		
Indeterminate	5		
Positive	783		
Her-2		0.07531	.9630
Negative	639		
Equivocal	10		
Positive	111		
Days to death		248.3	**.000**
<1324	100		
≥1342	102		
Stage		41.01	**.000004972**
I	111		
IA	90		
IB	7		
II	6		
IIA	398		
IIB	296		
III	2		
IIIA	173		
IIIB	29		
IIC	70		

## 4 Discussion

TIMP2 is a naturally secreted and 21 kDa unglycosylated protein, which could inhibit the activity of MMPs through binding in a 1:1 stoichiometric ratio to MMPs, like MMP-2 and MMP-9.^[11,12]^ Numerous researches showed that TIMP2 was involved in cell proliferation, apoptosis, angiogenesis, invasion, metastasis, and so on.^[13,14]^ However, the exact roles and the patterns of expression of TIMP2 in breast cancer remained unclear. In this research, we systematically explored the expression patterns, clinical characteristics, correlations, and prognostic values of TIMP2 in breast cancer.

Oncomine databases determined that the expression of TIMP2 was down-regulated in breast cancer, and was related to different types of breast cancer. Additionally, it was significantly down-regulated in breast carcinoma, ductal breast carcinoma, invasive breast carcinoma, invasive lobular breast carcinoma, invasive ductal and lobular breast carcinoma, mucinous breast carcinoma, and medullary breast carcinoma. Chien et al found that the enhancer of zeste homolog 2 could promote metastasis in triple-negative breast cancer through regulating TIMP2 and MMPs.^[12]^ Wang et al showed that TIMP2 gene polymorphism (rs2277698) was related to breast cancer risk in a Han Chinese cohort.^[15]^ Zhang et al discovered long noncoding RNA FENDRR could suppress the progression via regulating miR-761/TIMP2 in non-small-cell lung cancer.^[16]^ More researches are needed to discover the molecular mechanisms of TIMP2 in various types of breast cancer.

We further investigated the expression of TIMP2 among different clinical parameters in breast carcinoma through bc-GenExMiner software, such as age, hormone status, and subtypes. We could evaluate the importance of TIMP2 in breast cancer according to clinical characteristics. However, it needs more clinical data from various centers. We also confirmed the prognosis by UCSC Xena, which showed a significant difference in breast cancers. Therefore, TIMP2 might be a newly identified diagnosis and prognosis molecular in breast cancer. In osteosarcoma, miR-93 directly targeted TIMP2 which was associated with poor overall survival and prognosis.^[17]^ In co-expression network assay, we showed TIMP2 correlated with the regulation of EMT (SNAI2 and FN1), cell adhesion (integrin family, COL1A2, COL6A3, and COL3A1), and angiogenesis (MMP2, VEGFC), which we had not investigated further and warranted further study. However, the molecular mechanism had not been performed in breast cancer and we estimate that TIMP2 might be a diagnostic and potential therapeutic target for breast cancer even for other solid human cancers. In our previous study, we found serum exosomes with microRNAs, DNA, and protein could be a new biomarker in cancer.^[18]^ Also, serum exosomes from breast cancer may carry with TIMP2 as a new biomarker.

In conclusion, TIMP2 could be considered by a potential and promising target for novel therapeutics and biomarkers for breast cancer. Accompanying this discovery, we also need to perform more research and further understand the experimental molecular mechanisms of TIMP2 to validate in vitro and in vivo.

## Acknowledgments

This research was supported by the National Key Research and Development Program of China (No. 2016YFC0905900), National Natural Science Foundation of China (No. 81872365), and grants from the Jiangsu Provincial Key Research Development Program (No.BE2019731).

## Author contributions

Jin-Hai Tang and Dan-Dan Wang conceived and designed the experiments. Su-Jin Yang and Wen-Xiu Xu analyzed the data. Fei Deng contributed analysis tools. Wen-Quan Chen wrote the paper.

**Conceptualization:** Wen-Xiu Xu.

**Data curation:** Su-Jin Yang.

**Formal analysis:** Su-Jin Yang.

**Investigation:** Fei Deng.

**Methodology:** Fei Deng.

**Resources:** Wen-Xiu Xu.

**Writing – original draft:** Wen-Quan Chen, Dan-Dan Wang, Jin-Hai Tang.

**Writing – review & editing:** Dan-Dan Wang, Jin-Hai Tang.
